# An arcane presentation of pustular psoriasis in pregnancy: case report

**DOI:** 10.11604/pamj.2022.43.104.33237

**Published:** 2022-10-26

**Authors:** Arun Raja, Pragadeesh Palaniappan, Devika Sankar, Krishna Prasanth Baalann

**Affiliations:** 1 Department of Community Medicine, Sree Balaji Medical College and Hospital, Bharath Institute of Higher Education and Research (BIHER), Chennai, India,; 2 Department of Obstetrics and Gynecology, Government Theni Medical College and Hospital, Theni, India

**Keywords:** Pustular psoriasis of pregnancy, impetigo herpetiformis, generalized pustular psoriasis, case report

## Abstract

Pustular psoriasis of pregnancy (PPP) also known as impetigo herpetiformis is a well-described dermatosis of pregnancy characterized by the fatal progression of disease for both the mother and the foetus if left untreated. A 28-year-old G2P1L1 pregnant mother at 28 weeks of gestation, came to outpatient department (OPD) with complaints of scaly skin lesions all over her body along with fever, nausea and generalised weakness. On examination, there were erythematous scaly patches in the trunk, back, hands and legs accompanied by formation of pustules in the periphery of the lesions. Histopathological examination was consistent with pustular psoriasis. Patient was managed with prednisolone (40 mg/day which was later tapered). Serial antenatal visits and ultrasounds were done to monitor the health of the mother and foetal growth. Under the support of obstetrician, patient delivered a healthy female baby through caesarean section under general anaesthesia. Her lesions persisted in the postpartum period, which later started reducing gradually.

## Introduction

Pustular psoriasis of pregnancy (PPP), also known as impetigo herpetiformis, is a well-known pregnancy dermatosis distinguished by a deadly disease prognosis for both the mother and the baby if left untreated. This disease is usually thought to be a variant of generalized pustular psoriasis (GPP) and hence, it is also referred to as generalized pustular psoriasis of pregnancy (GPPP). In the late nineteenth century, the first occurrences of PPP were documented in the literature. Impetigo herpetiformis is indeed considered as a misnomer because PPP is neither connected to bacterial colonisation nor the herpes simplex virus [1].

Owing to the importance of early recognition and treatment, most experts agree on the classification of PPP as a dermatosis of pregnancy [2]. Though the timing of onset can range from the first trimester to the immediate post-partum period, PPP typically appears in the 3^rd^ trimester and resolves rapidly after delivery [3]. However, in subsequent pregnancies, it can recur often at an earlier stage. Pustular psoriasis of pregnancy has also been reported with oral contraceptive use and the menstrual cycle [4]. Women who develop pustular psoriasis of pregnancy may or may not have a personal or family history of psoriasis.

## Patient and observation

**Patient information:** a 28-year-old G2P1L1 pregnant mother at 28 weeks of gestation, came to OPD with complaints of scaly skin lesions all over her body along with fever, nausea, and generalized weakness. The patient was a known case of psoriasis from childhood on intermittent treatment with steroids. She was apparently normal a few months back after which, she was tapered on steroids as she got pregnant. The patient had similar history of exacerbation of lesions during first pregnancy which was mild in nature comparatively and was managed with topical corticosteroids. In the second pregnancy, the exacerbation of lesions occurred during the second trimester, when the lesions started arising in the trunk initially, which later spread rapidly to other parts of the body.

**Figure 1:**
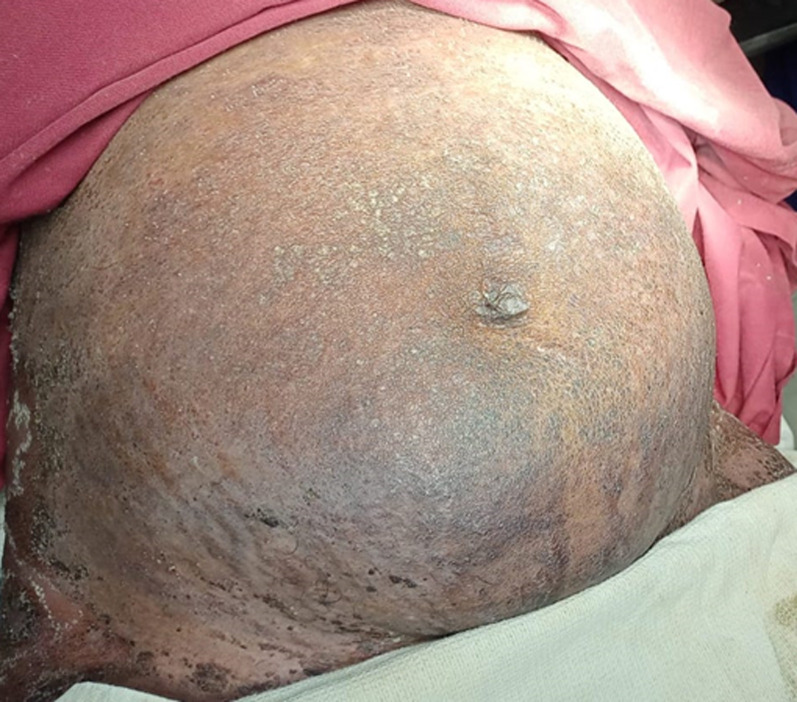
erythematous scaly lesions in the trunk, with pustules in the periphery

**Figure 2:**
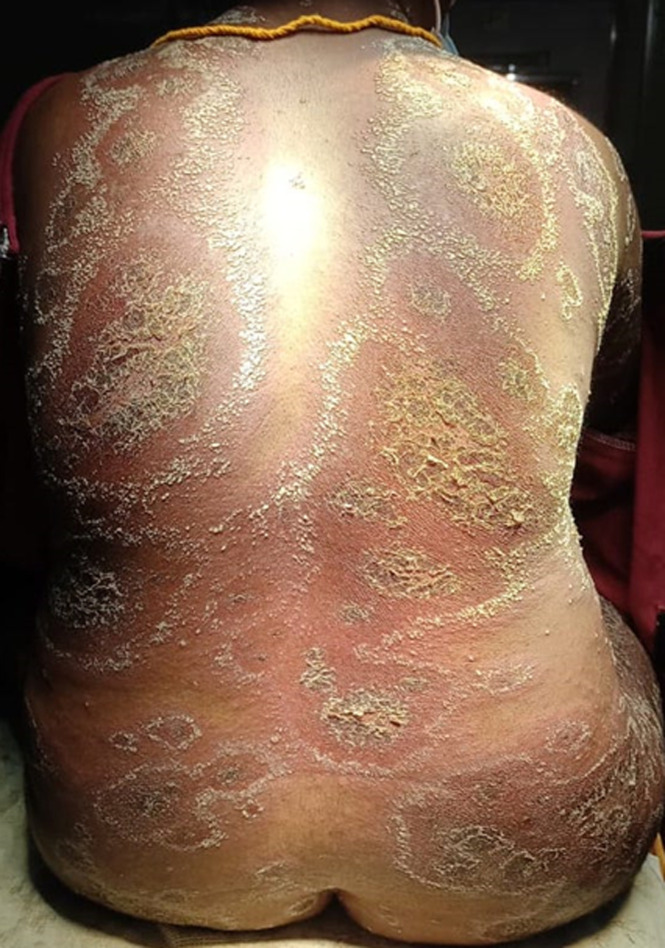
erythematous scaly lesions in the back, with pustules in the periphery

**Clinical findings:** on examination, there were erythematous scaly patches in the trunk (Figure 1), back (Figure 2), hands and legs accompanied by the formation of pustules in the periphery of the lesions. Along with the skin lesions, she developed fever, nausea and generalized weakness. Her vitals were stable. A dermatology opinion was obtained and the patient was provisionally diagnosed as pustular psoriasis of pregnancy.

**Diagnostic assessment:** all necessary blood investigations were done which revealed, Hb-9.8 g/dl, total count of 14,440/mm^3^ with predominant neutrophils, elevated ESR-74mm/h and CRP-65 (<5). Serum calcium was found to be normal and there were no electrolyte abnormalities. Kidney, liver and thyroid function tests were normal. Culture of pustules and blood did not pick-up any bacterial infection. Direct immunofluorescence was found to be negative.

**Figure 3:**
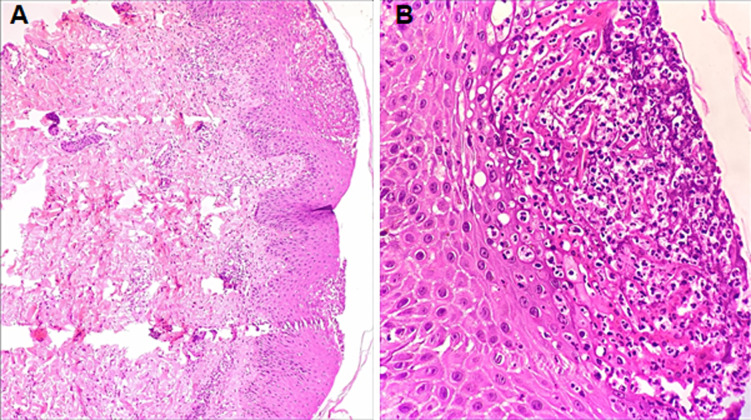
(A,B) epidermis with acanthosis and intraepidermal neutrophils and eosinophils, multiple pustules filled with neutrophils in the stratum corneum, spongiform pustule of Kogoj in the epidermis, mild oedema with many perivascular and interstitial neutrophils and eosinophils in the upper epidermis

**Diagnosis:** histopathological examination was consistent with pustular psoriasis, which showed, epidermis with acanthosis and intraepidermal neutrophils and eosinophils, multiple pustules filled with neutrophils in the stratum corneum, spongiform pustule of Kogoj in the epidermis, mild oedema with many perivascular and interstitial neutrophils and eosinophils in the upper epidermis (Figure 3).

**Therapeutic interventions:** patient was started on prednisolone 40 mg/day which was later tapered to 30 mg/day as the patient responded well. Antibiotic coverage (cephalosporine) was given to prevent any secondary infection. Serial antenatal visits and ultrasounds were done to monitor the health of the mother and foetal growth. Under the support of obstetrician, patient delivered a healthy female baby through caesarean section under general anaesthesia.

**Figure 4:**
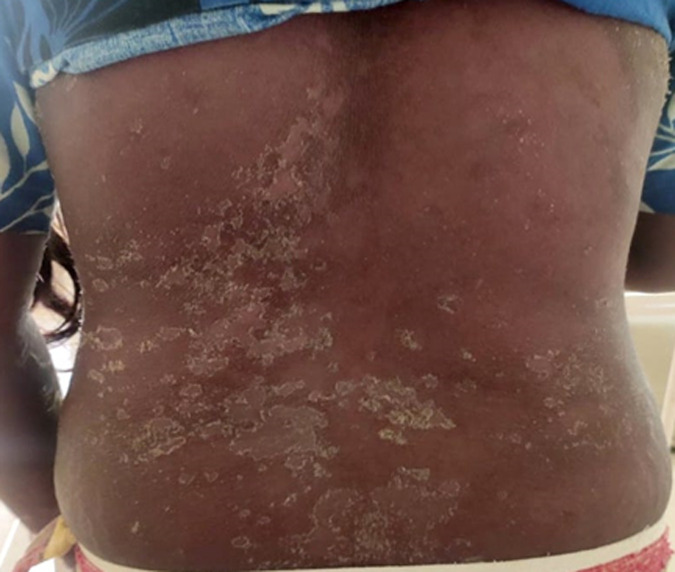
lesions during the postpartum period after healing gradually

**Follow-up and outcome of interventions:** her lesions persisted in the postpartum period, which later started reducing gradually (Figure 4). Steroids were tapered further and all necessary supportive measures were provided along with health education to the patient as well as the family members.

## Discussion

Von Hebra *et al*. first described the name impetigo herpetiformis (IH) in 1872 after reporting five pregnant women with pustular clustered lesions with inflammatory nature and crust evolution, all of which evolved into foetal fatalities, in addition to four maternal deaths [5]. Later in 1910, Von Zumbusch, first described the term, generalized pustular psoriasis (GPP) [6]. Currently, it is being termed as pustular psoriasis of pregnancy (PPP) by many authors, owing to the fact that the pustules are sterile and do not have any viral aetiology. Furthermore, there has been emerging evidence in recent years that this disorder is most likely a variation of GPP that flares up in response to a variety of triggers, such as metabolic disturbances, systemic steroid withdrawal, and pregnancy [7].

**Pathophysiology:** owing to the low incidence of pustular psoriasis of pregnancy, the pathophysiology is even more uncertain than in other forms of pustular psoriasis. There have been several reports of genetic mutations (IL36RN) predisposing patients to developing GPP [8]. To date, 17 pathogenic mutations in IL36RN have been reported in GPP or other inflammatory pustular skin disorders [9]. Common factors associated with PPP discussed is the literature includes, personal or family history of psoriasis, low serum vitamin D, increasing levels of progesterone during the last trimester of pregnancy, hypocalcaemia, and lower levels of elafin, an epidermal skin-derived antileukoproteinase [3,7,10]. The disruption in elastase activity was considered to be a major contributor in pustule formation in PPP (impetigo herpetiformis) patients. They went on to say that this rationale could also apply to GPP patients, bolstering the theory that PPP is a GPP variant [10].

There have also been reports of possible triggers associated with development of PPP such as, hormonal contraception, increased stress levels, seasonal changes, concurrent bacterial infections, certain medications [7] and hypoparathyroidism. There have also been incidents, where there is acute flare up or worsening of GPP during pregnancy. Previous research has suggested that around 55% of women with psoriasis see an improvement in their psoriasis symptoms during pregnancy, while the other 45% experience either no change or a worsening of symptoms [11].

**Treatment:** treatment of PPP initially includes topical steroids in mild cases. Fluids and electrolytes maintenance, especially calcium, and iron and vitamin D must be considered. In-spite of guidelines for the management of GPP being evolved continuously, immunosuppression have always been the mainstay of management. In 2011, Robinson *et al*. [12] in their study described a protocol for management of GPP during pregnancy. The first line management includes topical agents (for more limited disease or as an adjuvant treatment), oral corticosteroids, cyclosporin, biologic agents (infliximab). Second line management includes narrow band ultraviolet-B (UVB), oral psoralens long wave ultraviolet radiation (PUVA).

Corticosteroids have always been one of the major drug in the management of GPP. But caution should be taken in case of PPP. Mild cases can be treated with low dose prednisolone (15-30 mg/day). Whereas, in severe cases as high as 60-80 mg/day can be used. The use of steroids in PPP is more effective when it is combined with cyclosporine. Though cyclosporin has been considered as a category C drug in pregnancy, it is being used as a first line drug in management of moderate to severe disease as there have been several published cases [13] reporting the successful use of cyclosporine (2-5 mg/day) in pregnant patients with no adverse fetal outcomes related to medication use. Although, cyclosporine is thought to be a safer drug for managing PPP, it should be avoided while breastfeeding as there is not much clear information about the amount of exposure to the infant occurring through breastmilk.

Biologic agents (primarily infliximab) has been successfully used to treat the severe form of GPP [14]. But they belong to pregnancy category B. There are only a few reports of infliximab use for the treatment of PPP [14,15]. Even if used, it has been recommended not to use after 30 weeks of pregnancy as there is higher chances for placental transfer of IgG antibodies. Among the second line management, narrow band UVB is preferred more when comparing to PUVA, as the former is more safer during pregnancy. Vun YY *et al*. [16] reported the first case of PPP, which was successfully treated by narrow band UVB. However, in postpartum period PUVA is used topically [12,17].

Though the pustules are sterile, it has been recommended to use systemic antibiotic concurrently with other drugs for the prevention of secondary infection. The preferred antibiotic of choice is cephalosporins [7,18]. PPP can lead to serious complications in fetal health, which include intrauterine growth restriction, premature rupture of membranes, miscarriage, and even stillbirth. If the pregnant woman is having systemic signs and symptoms in association with PPP, careful and continuous monitoring of mother and fetus will be necessary, owing to the increased risk of placental insufficiency, foetal abnormalities, and foetal death [19].

Other treatments and considerations include use of emollients (or) moisturizers, reduction of stress during pregnancy (enough sleep, exercise, yoga/meditation, etc.). In case of severe disease, when the maternal health is being compromised, induction of labour can be preferred as a therapeutic approach [20]. During prenatal as well as postnatal visits, the patients and family members should indeed be offered psychological help. Being known that, PPP can occur during an earlier phase with worse prognosis in the subsequent pregnancies, the healthcare provider must give counselling regarding future pregnancy plans and contraceptive methods to the patients.

**Differential diagnoses:** pustular psoriasis, dermatitis herpetiformis, acute generalised exanthematous pustulosis, polymorphic eruption of pregnancy, atopic eruption of pregnancy, erythema multiforme, pustular sub-corneal dermatosis and gestational pemphigoid.

## Conclusion

The challenges faced by the healthcare professionals with PPP is that, it is relatively a rare diagnosis, which has an unknown and controversial etiopathogenesis, that complicates both maternal and foetal health. Early diagnosis and appropriate treatment play a crucial role in the prognosis of the disease and the outcome of pregnancy. Clear clinical description and biopsy confirmation are mandatory as this disease can resemble any other pustular dermatoses. Though, understanding the genetic involvement and the pathophysiology are not routinely helpful in the diagnosis, they pave the way for future treatment modalities. For improving the quality of life of pregnant mothers and to have a favourable outcome in the foetal health, the obstetricians, dermatologists and the paediatricians must work together to tackle the challenges of pustular psoriasis of pregnancy.
